# Genome-Wide Identification and Expression Analysis of the PEBP Gene Family in *Cymbidium sinense* Reveals *CsFTL3* as a Floral Inhibitor

**DOI:** 10.3390/plants15020252

**Published:** 2026-01-13

**Authors:** Wei Zhu, Chunfeng Chen, Yonglu Wei, Yanmei Sun, Jie Gao, Jie Li, Qi Xie, Jianpeng Jin, Chuqiao Lu, Genfa Zhu, Fengxi Yang

**Affiliations:** Guangdong Key Laboratory of Ornamental Plant Germplasm Innovation and Utilization, Environmental Horticulture Research Institute, Guangdong Academy of Agricultural Sciences, Guangzhou 510640, China; zhuwei0923@126.com (W.Z.); ccf18864835013@stu.scau.edu.cn (C.C.); weiyonglu@gdaas.cn (Y.W.); 2019220006@njau.edu.cn (Y.S.); gaojie@gdaas.cn (J.G.); lijie@gdaas.cn (J.L.); xieqi@gdaas.cn (Q.X.); jinjianpeng@gdaas.cn (J.J.); luchuqiao@gdaas.cn (C.L.)

**Keywords:** orchid, flowering transformation, PEBP family, flowering time, VIGS

## Abstract

This study comprehensively characterizes the PEBP gene family in *Cymbidium sinense*, an orchid with a prolonged vegetative phase that limits its industrial production. Genome-wide analysis identified six CsPEBPs, classified into *FT*-like, *TFL1*-like, and *MFT*-like subfamilies. Evolutionary, gene structure, and collinearity analyses revealed both conservation and lineage-specific diversification of these genes. *CsFTL3*, a distinctive FT-like member, displayed notably high expression during the bud undifferentiated stage, followed by a sharp downregulation upon floral initiation. Functional studies identified *CsFTL3* as a key floral repressor. Heterologous overexpression in *Arabidopsis* delayed flowering time from 32.0 days (wild-type) to 63.0–75.3 days (transgenic) and increased rosette leaf number from 12.6 to 33.0–34.5, while its knockdown via virus-induced gene silencing (VIGS) in *C. sinense* accelerated floral bud development and upregulated flowering-promoter genes. Phylogenetically, CsFTL3 falls within the flowering repressor FT-I clade, and multiple sequence alignment identified critical amino acid substitutions (Y134S, W138L, Q140E) that likely underpin its functional divergence from typical flowering promoters. Furthermore, promoter analysis revealed an enrichment of light-, hormone-, and stress-responsive cis-elements, and its expression was modulated by gibberellin (GA), abscisic acid (ABA), and low-temperature treatments. Predicted protein–protein interaction and transcriptional regulatory networks provide preliminary insights into its complex regulation. We conclude that *CsFTL3* acts as a crucial floral inhibitor, integrating environmental and endogenous cues to repress flowering. These findings offer fundamental insights into the molecular mechanisms of flowering in orchids and provide a valuable genetic resource for molecular breeding programs aimed at achieving precise flowering time control.

## 1. Introduction

*Cymbidium sinense*, a member of the Orchidaceae family, is an economically and ornamentally valuable plant. It is widely appreciated as an ornamental flower due to its elegant floral morphology and distinctive fragrance [1,2]. The floral scent also contains valuable sesquiterpenoids, such as farnesol, which are important natural aromatic compounds with promising applications in the flavor and fragrance industry [3,4]. In addition, the plant has medicinal properties and has traditionally been used as a tonic and for treating various conditions, including chronic diseases, dizziness, eye disorders, and burns [5]. As a high-end flower, the industrial value of *C. sinense* largely depends on traits such as flower yield, quality (including fragrance composition and floral form), and propagation efficiency [1]. To optimize these traits, the regulation of flowering time is a crucial technical factor for achieving year-round supply, enhancing commercial value, and strengthening market competitiveness [6]. However, its narrow natural flowering window, prolonged vegetative growth phase, and complex flowering mechanism significantly constrain precise flowering time control and molecular breeding progress [7,8]. Furthermore, the ornamental value of *C. sinense* is intrinsically linked to its unique orchid floral architecture, characterized by a specialized labellum and a fused reproductive column, as well as its multi-flowered racemose inflorescences [1]. The development of such complex floral structures requires precise genetic coordination with the flowering timing signals. This genetic coordination involves conserved MADS-box transcription factors, such as *AP3*/*DEF*-like, *AGL6*-like, and *SEP*-like genes, known to specify orchid floral organs [9,10]. Therefore, a systematic understanding of the molecular regulatory mechanisms underlying floral transition in *C. sinense* is essential. This knowledge is fundamental to achieving precise flowering time control, improving floral and aromatic product quality, and advancing germplasm innovation, as well as efficient propagation technologies.

Plant flowering timing is coordinated through the integration of endogenous signals and external environmental cues [11,12]. Within this complex regulatory network, the Phosphatidylethanolamine-Binding Protein (PEBP) gene family serves as a central component of florigen signaling, functioning as a key hub that integrates multiple flowering pathways including photoperiod, vernalization, and hormonal regulation [13]. All PEBP family members contain two highly conserved short motifs, DPDxP and GxHR, which are potentially involved in forming the ligand-binding pocket [14]. In higher plants, the PEBP family is evolutionarily conserved and is divided into three functionally distinct subfamilies: FLOWERING LOCUS T (*FT*)-like (e.g., *FT*, *TSF*; promoting flowering), TERMINAL FLOWER 1 (*TFL1*)-like (e.g., *TFL1*, *BFT*, *CEN*; inhibiting flowering and maintaining vegetative growth), and MOTHER OF *FT* AND *TFL1* (*MFT*)-like (primarily involved in processes such as seed germination) [15]. In *Arabidopsis thaliana*, FT protein is synthesized in leaf vascular tissues, transported to the shoot apical meristem (SAM) facilitated by FT-INTERACTING PROTEIN (FTIP), and subsequently forms a ternary complex with the transcription factor FD and 14-3-3 proteins [16,17]. This complex activates floral meristem identity genes such as *AP*1 and *LFY*, thereby initiating flowering [18]. Conversely, the TFL1 protein suppresses the floral transition through a similar protein interaction competition mechanism [19]. The precise expression of these key floral regulators is orchestrated by complex transcriptional networks, where the architecture of cis-regulatory elements in their promoters plays a critical role in determining their spatiotemporal specificity [20].

The rapid progress in plant genome sequencing has enabled the systematic identification of PEBP family members across a wide range of species. These include cereal and oilseed crops such as rice [21], potato [22], and *Brassica napus* [23]; horticultural crops including *Solanum lycopersicum* [24], pineapple [25], and pear [26]; and economically important tree species like *Macadamia integrifolia* [27], *Castanea mollissima* [28], and *Phyllostachys heterocycla* [29]. To further clarify the evolutionary mechanisms of this gene family—including its origin, duplication, and functional differentiation—comprehensive family-level phylogenetic studies have been conducted in groups such as Sapindaceae [30], Rosaceae [31], Juglandaceae [32], and Cucurbitaceae [33].

Within the Orchidaceae family, the PEBP gene family has been identified in several species, including *Phalaenopsis* hybrids [34], *Dendrobium* [35,36], and *C. ensifolium* [37], providing a basis for functional studies. Subsequent functional studies have revealed evolutionary conservation and functional complexity among orchid PEBP genes. This functional conservation is evidenced by the preserved flowering-promoting function of *Cymbidium sinense CsFT* when heterologously overexpressed in *Arabidopsis* [38]. Functional complexity, however, manifests at multiple levels. Firstly, within the same genus (*Dendrobium*), *DhFT* and *DhTFL1* exhibit antagonistic expression patterns in response to GA treatment, thereby working coordinately to fine-tune flowering time [35]. Additionally, PEBP members demonstrate functional specificity in floral organ development across different *Dendrobium* species [36]. In *Phalaenopsis*, notable functional divergence has been observed among family members: while heterologous expression of *PhFT1*, *PhFT3*, *PhFT5* and *PhMFT* promotes flowering, that of *PhFT6* unexpectedly inhibits it, suggesting the potential existence of extensive antagonistic regulatory networks within this gene family [34].

However, a systematic understanding of the PEBP gene family in *C. sinense* remains elusive. Specifically, the genomic composition and evolutionary relationships of its PEBP members have not been determined. More importantly, no *FT*-like member has been functionally demonstrated to act as a floral repressor in *C. sinense*. This gap in knowledge significantly limits our ability to explain how the prolonged vegetative phase of *C. sinense* is regulated at the molecular level, particularly via the regulatory roles of this key gene family. Flowering in *Cymbidium* is modulated by a complex interplay of environmental and hormonal signals, such as environmental cues (e.g., low-temperature and light), nutrient availability, and exogenous hormones like gibberellin (GA, which promotes bud elongation) and abscisic acid (ABA, which extends dormancy) [39,40,41,42]. Consequently, a key unresolved question is whether these diverse cues converge to regulate flowering time in *C. sinense* by modulating its PEBP genes. Addressing this question is essential for achieving precise control over flowering in this valuable orchid species.

Therefore, this study aimed to systematically elucidate the molecular mechanisms regulating flowering in *C. sinense* by focusing on the phylogenetically distinctive *FT*-like gene, *CsFTL3*. We hypothesized that *CsFTL3* functions as a floral repressor, integrating environmental cues to delay the vegetative-to-reproductive transition. To test this hypothesis, we first conducted a genome-wide identification and expression profiling of the entire PEBP gene family in *C. sinense*. Subsequently, we specifically investigated *CsFTL3* through gene cloning and phylogenetic analysis to assess its evolutionary position and heterologous overexpression in *Arabidopsis* to examine its effect on flowering time and related gene expression, subcellular localization, and analysis of its response to hormone and low-temperature treatments. Furthermore, we predicted its protein interaction partners and upstream transcriptional regulators to preliminarily map its regulatory network. Collectively, this multifaceted approach was designed to validate the inhibitory role of *CsFTL3* and provide insights into its molecular function within the flowering regulatory framework of *C. sinense*.

## 2. Materials and Methods

### 2.1. Plant Materials and Growth Conditions

In this study, *C. sinense* ‘Xiao Xiang’ plants were grown in the Orchid Resource Garden of Environmental Horticulture Research Institute, Guangdong Academy of Agricultural Sciences. Plants were maintained under conventional water and fertilizer with regular repotting. Various organs (root, stem, leaf, flower, fruit, and flower buds) were collected, flash-frozen in liquid nitrogen, and stored at −80 °C until RNA extraction. For genetic transformation, *Arabidopsis thaliana* ecotype Columbia (Col-0) was used as the wild type.

To examine the effects of abiotic stress on the expression of PEBP family genes in *C. sinense*, we applied exogenous hormone sprays and low-temperature treatments. Uniformly grown potted seedlings were subjected to the following experimental treatments: spraying 100 μM GA_3_ or 100 μM ABA, respectively (with distilled water spray as control), or exposure to 4 °C cold stress (with normal temperature growth as control). Each treatment included at least three biological replicates. Flower bud samples were collected at 0, 4, 8, and 12 h after hormone application, and at 0, 2, and 4 days after cold treatment. All samples were immediately flash-frozen in liquid nitrogen and stored at −80 °C for subsequent total RNA extraction.

### 2.2. Identification and Analysis of the PEBP Gene Family in C. sinense

The PEBP gene family in *C. sinense* was identified through a systematic search of its published genome [43,44]. First, a BLASTP search was performed using the conserved PEBP domain (PF01161) as the query (E-value cutoff ≤ 1 × 10^−5^). Additionally, to ensure comprehensiveness, a complementary search was conducted using HMMER (version 3.3.2) with the corresponding hidden Markov model (HMM) profile from the Pfam database. The candidate sequences identified by both methods were combined, and redundant entries were removed. The presence of the conserved PEBP domain in all candidate proteins was further verified using the NCBI CD-search tool. The nucleotide and amino acid sequences of the confirmed CsPEBPs were then downloaded for subsequent analysis. According to the naming rules of *A. thaliana*, the identified CsPEBPs were named and classified. Physical and chemical properties such as theoretical isoelectric point (PI), molecular weight (MW) and grand average of hydrophilicity (GRAVY) of amino acid sequence of CsPEBPs were identified by using Protparam tool on Expasy (https://web.expasy.org/protparam/ (accessed on 25 March 2025)) online website [45,46]. The subcellular localizations of CsPEBPs were predicted using Cell-PLoc (http://www.csbio.sjtu.edu.cn/bioinf/Cell-PLoc/ (accessed on 25 March 2025)) [47].

### 2.3. Phylogenetic Analysis of CsPEBPs

A phylogenetic tree was constructed with MEGA 12.0 (Pennsylvania State University, State College, PA, USA) based on 150 PEBP protein sequences from 16 species, using the Neighbor-Joining (NJ) method. The parameters of the phylogenetic tree were 1000 bootstrap replicates and default for other parameters [32,48]. The online software iTOL (v7.2) was applied to beautify the phylogenetic tree (https://itol.embl.de/ (accessed on 13 April 2025)). All sequences used in this analysis are listed in Table S1.

### 2.4. Analysis of Gene Structure and Conserved Motifs

The exon-intron structures of the identified CsPEBPs were retrieved from the *C. sinense* genome GFF file and visualized using TBtools-II (v2.310) [49]. Conserved motifs in each identified CsPEBP protein were identified using MEME Suite (v5.5.8) (https://meme-suite.org/meme/tools/meme (accessed on 4 July 2025)).

### 2.5. Cis-Element Identification and Protein-Interaction Prediction

The 2000 bp sequence upstream of the initiation codon (ATG) of CsPEBPs was extracted from the *C. sinense* genome. *Cis*-acting elements of all CsPEBPs were analyzed and identified online by PLANTCARE (http://bioinformatics.psb.ugent.be/webtools/plantcare/html/ (accessed on 4 July 2025)) [50]. The results were visualized using TBtools-II (v2.310).

Potential protein–protein interaction for CsFT and CsFTL3 were predicted using the STRING (v12.0) (https://string-db.org/ (accessed on 4 July 2025)) based on their amino acid sequences. The protein–protein interaction (PPI) network was drawn by Cytoscape (v3.10.3).

Specific primers (Table S2) were designed based on the promoter sequence of *CsFTL3*. The corresponding promoter fragment was then amplified via PCR using genomic DNA extracted from flower buds of the *C. sinense* ‘Xiao Xiang’ as the template. Taking *Phalaenopsis*, rice and *Arabidopsis* as reference, possible upstream TFs regulating *CsFTL3* were identified using the PlantRegMap database (https://plantregmap.gao-lab.org/binding_site_prediction.php (accessed on 7 July 2025)). Then, TBtools-II (v2.310) was used to screen homologous TFs in *C. sinense* genome. The network of upstream TFs was drawn by Cytoscape (v3.10.3) [46]. Transcriptome data of upstream TFs at different flower development stages were obtained from our previously published transcriptome study [42]. The heat map of potential TFs was visualized using TBtools-II (v2.310).

### 2.6. Chromosomal Location and Collinearity Analysis

Chromosomal locations of the *C. sinense* PEBP gene family were extracted from its genome sequence and General Feature Format (GFF) file, and were mapped and visualized using TBtools-II (v2.310). Genome sequences for *C. ensifolium*, *P. equestris*, *Apostasia shenzhenica*, *Oryza sativa*, and *A. thaliana* were downloaded from the Ensembl Plants database (http://plants.ensembl.org/index.html (accessed on 12 July 2025)). Collinearity files between each species pair were obtained using Multiple Collinearity Scan toolkit X version (MCScanX) [2,51]. The collinearity analysis diagrams were subsequently generated with TBtools-II (v2.310).

### 2.7. Gene Expression Analysis by qRT-PCR and RNA-Seq

Total RNA was extracted from each sample using the FastPure Universal Plant Total RNA Isolation Kit (Vazyme, Nanjing, China) following the manufacturer’s instructions. RNA concentration was measured using a NanoDrop 2000 spectrophotometer (Thermo Fisher Scientific, Waltham, MA, USA). cDNA was synthesized using the HiScriptIIQ RT SuperMix for qPCR (+gDNA wiper) kit (Vazyme, Nanjing, China). Quantitative real-time PCR (qRT-PCR) was performed using the SYBR Green qPCR Master Mix (Thermo Fisher Scientific, USA) with three independent biological replicates per sample. *CsActin* and *AtActin* were used as internal reference genes for *C. sinense* and *A. thaliana*, respectively. Gene-specific primers (Table S2) were designed using NCBI Primer-BLAST (https://www.ncbi.nlm.nih.gov/tools/primer-blast/index.cgi?LINK_LOC=BlastHome (accessed on 5 May 2025)). Relative expression levels were calculated using the 2^−ΔΔCt^ method [52].

To provide an initial expression profile of the CsPEBP family, publicly available RNA-Seq data (NCBI BioProject PRJNA743748) were analyzed. This dataset, which included three biological replicates per condition, was used to calculate FPKM (Fragments Per Kilobase of exon per Million mapped reads) values for visualization and descriptive trend analysis (Figure S1). All quantitative comparisons and functional inferences related to gene expression are based on the qRT-PCR validation data presented in the main figures.

### 2.8. Cloning, Sequence Alignment and Phylogenetic Tree Analysis

The full-length CDS of *CsFTL3* was obtained from the *C. sinense* genome database [43], and specific primers were designed (Table S2). PCR amplification was performed using petal cDNA of *C. sinense* ‘Xiao Xiang’ as template with 2× Phanta Flash Master Mix (Dye Plus) (Vazyme, Nanjing, China). The reaction protocol was as follows: initial denaturation at 98 °C for 30 s; 35 cycles of denaturation at 98 °C for 10 s, annealing at 55 °C for 5 s, and extension at 72 °C for 5 s; final extension at 72 °C for 1 min. The PCR products were purified with the FastPure Gel DNA Extraction Mini Kit (Vazyme, Nanjing, China), and then ligated and transformed into DH5α competent *E. coli* cells using the 5 min TA/Blunt-Zero Cloning Kit (Vazyme, Nanjing, China). Positive clones were selected and sent for sequencing to Youkang Biotechnology Co., Ltd (Hangzhou, China). Amino acid sequences of CsFTL3 homologous proteins were retrieved from the NCBI database (Table S3). Multiple sequence alignment was conducted in DNAMAN (v9) (Lynnon Biosoft, San Ramon, CA, USA), and a phylogenetic tree was constructed with the neighbor-joining (NJ) method in MEGA12.0 (Pennsylvania State University, State College, PA, USA).

### 2.9. Subcellular Localization of CsFTL3 in C. sinense Protoplasts

The PAN580 vector was linearized with appropriate restriction enzymes. Homologous recombination primers were designed based on the *CsFTL3* CDS sequence (Table S2), and the target fragment was amplified using the cloning vector as a template. The recombinant vector PAN580-*CsFTL3*-GFP was constructed by inserting the target fragment into the linearized vector using the ClonExpress II One Step Cloning Kit (Vazyme, Nanjing, China). The verified recombinant plasmid was prepared on a large scale using the GoldHi EndoFree Plasmid Maxi Kit (Cowin Biotech, Taizhou, China). The recombinant plasmid was then transfected into *C. sinense* protoplasts via a previously established protoplast isolation and transient expression system (PTES) [53]. After incubation in darkness at 23 °C for 18–24 h, the protoplasts were stained with 50 µg/mL DAPI for 10 min and imaged using an LSM710 confocal laser scanning microscope.

### 2.10. Heterologous Overexpression of CsFTL3 in Arabidopsis

The pOCA30 vector was linearized with SalI and SacI. The target fragment was amplified using the *CsFTL3* cloning plasmid as a template with homologous recombination primers (Table S2) and purified. The linearized vector and the target fragment were assembled using an in-fusion cloning system to generate the pOCA30-*CsFTL3* construct. Positive clones were confirmed by colony PCR and Sanger sequencing. The pOCA30-*CsFTL3* plasmid was transformed into GV3101 *Agrobacterium* competent cells to obtain positive cultures. Wild-type *Arabidopsis* was transformed using the floral dip method. Transgenic positive plants were selected based on kanamycin resistance and further verified by PCR, with screening continued to the T3 generation. Both wild-type and transgenic plants were grown under identical conditions. The flowering time was recorded when the bolting stem reached 1 cm in height, and the number of rosette leaves at flowering was counted, and other phenotypic traits were observed.

### 2.11. Virus-Induced Gene Silencing (VIGS)

Functional validation was performed using VIGS. The pTRV vector system was employed, and the pTRV2 vector was linearized by double digestion with BamHI and KpnI. Based on the *CsFTL3* sequence, a 300-bp specific fragment was selected using an online design tool (https://vigs.solgenomics.net/ accessed on 24 July 2025), and homologous recombination primers were designed. The target fragment was obtained by PCR amplification. Subsequently, the fragment was ligated into the linearized pTRV2 vector via homologous recombination to construct the recombinant plasmid TRV2-*CsFTL3*. TRV2-*CsFTL3*, TRV1, and the empty TRV2 (negative control) were separately transformed into GV3101 *Agrobacterium* competent cells. Positive clones were selected and expanded. Bacterial suspensions were resuspended in infiltration buffer (100 µM AS, 10 mM MES, 10 mM MgCl_2_, pH 5.6) to an OD_600_ of 1.0. TRV1 and TRV2-*CsFTL3* suspensions were mixed in equal volumes as the treatment group, while TRV1 and empty TRV2 suspensions were mixed equally as the control group. The mixed suspensions were kept in darkness for 3 h. In mid-August, *C*. *sinense* ‘Xiao Xiang’ plants at the flower bud undifferentiation stage (S0) were selected for infection, with each clump containing about 4 shoots and each group comprising no fewer than 20 plants. Before infiltration, the pseudobulbs were gently pierced 3–5 times with a 1-mL syringe needle, avoiding the apical meristem. The pseudobulbs were then completely immersed in the mixed bacterial suspension and placed in a vacuum chamber. Vacuum infiltration was performed at 0.08 MPa for 10 min, followed by a slow release to atmospheric pressure over about 10 min. After infiltration, the bacterial solution was rinsed off, and the plants were repotted and maintained under routine greenhouse conditions. 30 days after infection, flower bud phenotypes were observed and samples were collected. The length of flower buds was measured with a ruler, and their internal structure was examined and photographed using a stereomicroscope. Positive silenced plants were identified by PCR, and the silencing efficiency of *CsFTL3,* together with the expression changes in other flowering-related genes, was detected by qRT-PCR. Primer sequences were listed in Table S2.

### 2.12. Data Statistics and Analysis

Data are expressed as the mean ± SEM of at least three biological replicates. Normality and homogeneity of variances were verified prior to analysis. Statistical analysis was performed using one-way analysis of variance (ANOVA) in SPSS software (SPSS, Chicago, IL, USA; v16.0). When the ANOVA indicated a significant overall effect (*p* < 0.05), post hoc multiple comparisons were conducted using the Least Significant Difference (LSD) test, with the significance level set at α = 0.05. In figures, significant differences are indicated by asterisks (* *p* < 0.05, ** *p* < 0.01) or distinct lowercase letters (*p* < 0.05). Data were processed in WPS Office Excel. Graphs were plotted using GraphPad Prism 9, and the final figures were assembled and annotated in Adobe Photoshop CS6.

## 3. Results

### 3.1. Identification and Analysis of the PEBP Gene Family in C. sinense

A genome-wide search identified six putative PEBP family genes in *C. sinense*. Phylogenetic analysis with *A. thaliana* homologs led to their designation as *CsFT*, *CsFTL1*, *CsFTL2*, *CsFTL3*, *CsTFL1* and *CsMFT* (corresponding gene IDs are provided in Table 1). Their basic protein characteristics, including amino acid (AA) length, molecular weight (MW), theoretical isoelectric point (PI), instability index, and grand average of hydropathicity (GRAVY), are summarized in Table 1.

The six CsPEBPs varied in length from 146 (CsMFT) to 181 (CsFTL2) AA, averaging 171 AA. Their genomic DNA (gDNA) sequences exhibited considerable length variation and ranged from 0.9 kp (CsTFL1) to 18.9 kp (CsFTL2). MW and isoelectric point (PI) ranged from 16.50 (CsMFT) to 20.63 (CsFTL2) kDa and 5.93 (CsFTL1) to 9.06 (CsTFL1), respectively. The instability index ranged from 43.37 (CsFT) to 63.67 (CsMFT), indicating that all six members were unstable proteins. The aliphatic index ranged from 73.42 (CsMFT) to 82.14 (CsTFL1). Interestingly, the GRAVY of all CsPEBPs was negative, which clearly showed their hydrophilicity. The prediction of subcellular localization showed that all CsPEBPs may have nuclear localization signals except CsMFT, which may be located in the cytoplasm. These molecular characterizations provide a foundation for investigating the biological functions of CsPEBPs.

### 3.2. Evolutionary and Phylogenetic Analysis of PEBP Genes in Different Species

**Figure 1:**
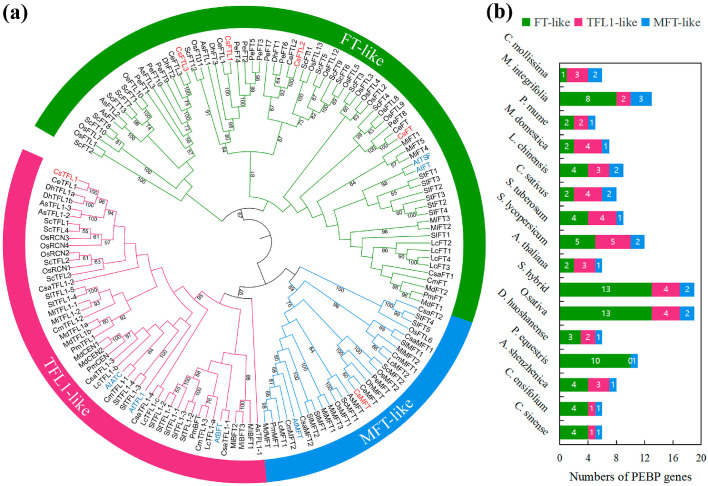
Phylogenetic analysis of the PEBP proteins from 16 different species. (**a**) A phylogenetic tree of 150 PEBP proteins from *Apostasia shenzhenica* (As), *A. thaliana* (At), *Castanea mollissima* (Cm), *Cucumis sativus* (Csa), *C. ensifolium* (Ce), *C. sinense* (Cs), *D. huoshanense* (Dh), *Litchi chinensis* (Lc), *Macadamia integrifolia* (Mi), *Malus domestica* (Md), *Oryza sativa* (Os), *P. equestris* (Pe), *P. mume* (Pm), *Saccharum hybrid* (Sc), *Solanum lycopersicum* (Sl) and *Solanum tuberosum* (St). The phylogenetic tree was divided into three groups, each group had a different color. The value in the branch represented the bootstrap values. (**b**) Statistics of three types of PEBP proteins in 16 plant species.

To assess the evolutionary relationship between plant PEBP genes, a phylogenetic tree was constructed using 150 PEBP amino acid sequences from 16 angiosperms (including monocotyledons and dicotyledons) (Figure 1a). According to the clustering results of Figure 1a and the classification criteria of PEBP gene family in *A. thaliana*, among 16 species, the phylogenetic tree was divided into three main branches, corresponding to three subfamilies: *FT*-like, *TFL1*-like and *MFT*-like. Therefore, we used green, red and blue to distinguish the three subfamilies of *FT*-like, *TFL1*-like and *MFT*-like. *FT*-like clade was the largest, comprising 54% of all PEBP genes analyzed. Notably, monocot species tended to harbor more PEBP genes than dicots. In *C. sinense*, the *FT*-like subfamily had the most *PEBP* genes, including *CsFT*, *CsFTL1*, *CsFTL2* and *CsFTL3*, while the *TFL1*-like and *MFT*-like subfamilies each had a PEBP gene, namely *CsTFL1* and *CsMFT*. *CsFT* was the orthologous gene of *AtFT* in *A. thaliana*, and the two genes were the first to get together, which indicated that *CsFT* may have a conservative function of promoting flowering in *C. sinense*. Previous studies have also preliminarily proved that *CsFT* can promote flowering by overexpressing it in *A. thaliana* [38]. *CsFTL3* evolved into a single branch in the *FT*-like subfamily, suggesting a possible functional divergence, potentially in its role in flowering regulation, which warranted further investigation. In addition, the dendrogram showed that PEBP had closely related orthologous with *C. sinense*, *C. ensifolium*, *P. equestris*, *D. huoshanense* and *A. shenzhenica* (such as CsTFL1/CeTFL1/DhTFL1a, b/AsTFL1-2 and AsTFL1-3), indicating possible functional conservation across these species.

To examine the evolution patterns of PEBP genes, we compared their numbers across 16 herbaceous and woody plants (Table S4; Figure 1b). There were some differences in the number of *PEBP* genes among 16 plant species, with the highest in *O. sativa* (19) and *S. hybrid* (19) and the lowest in *P. mume* (5). Compared with dicotyledons, monocotyledons generally had a lower *TFL1*-like subfamily gene ratio, and some (e.g., *P. equestris*) even lacked them entirely.

### 3.3. Gene Structure and Conserved Motifs of CsPEBPs

**Figure 2:**
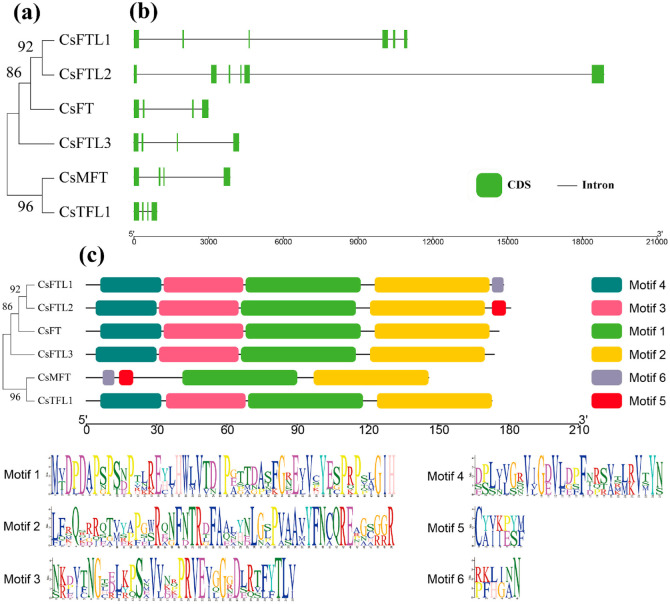
Phylogenetic tree, DNA structure and protein motif analysis of 6 CsPEBPs. (**a**) The evolutionary relationships of 6 CsPEBPs. (**b**) Exon-intron structure analysis of CsPEBPs. Green boxes and black lines represented exons and introns, respectively. (**c**) Motif distribution of CsPEBPs.

A phylogenetic tree of the six CsPEBP proteins grouped them into three subfamilies (Figure 2a). Analysis of exon-intron structures revealed that CsPEBP genes contain either 3 or 5 introns (corresponding to 4 or 6 exons), reflecting a relatively conserved gene architecture (Figure 2b). Among them, the *FT*-like subfamily members showed variation in intron number: *CsFTL1* and *CsFTL2* contained five introns, while *CsFT* and *CsFTL3* contained three, indicating structural diversification within this subfamily. Notably, *CsFTL1* and *CsFTL2* contained longer introns, which may influence transcriptional regulation and alternative splicing of genes.

Conserved motif analysis identified six motifs (motif1–motif6) ranging from 6 to 50 amino acids in length (Figure 2c). Each CsPEBP contained 4 to 5 conserved motifs. Except for CsMFT, the other members all contained four motifs from motif1 to motif4 in the same order, indicating high sequence conservation among these CsPEBPs. However, motif5 and motif6 were both present uniquely in CsMFT, suggesting it may have distinct functions. In the FT-like subfamily, motif6 and motif5 existed in CsFTL1 and CsFTL2, respectively, indicating that they may have other functions.

### 3.4. Chromosomal Location and Collinearity Analysis of CsPEBPs

**Figure 3:**
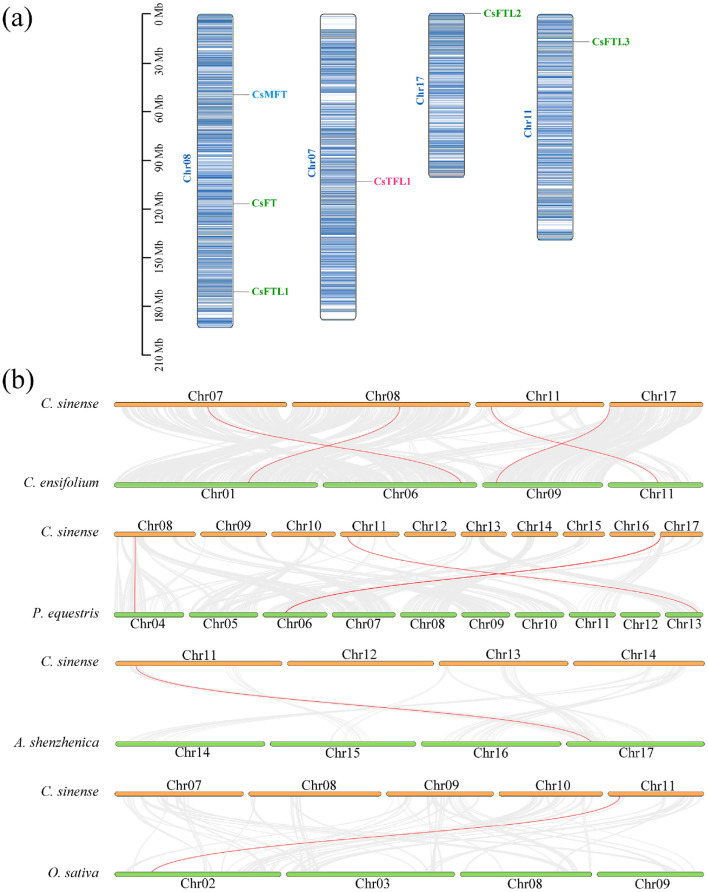
Chromosome location of CsPEBPs in *C. sinense* and analysis of its collinear relationship with PEBP genes in other species. (**a**) Chromosome location of CsPEBPs. (**b**) Collinearity analysis of the PEBP genes from *C. sinense* and four other species. Gray lines represent collinear blocks in *C. sinense* and other genomes, and red lines represent collinear gene pairs.

The six CsPEBPs were distributed across four chromosomes in *C. sinense*. Specifically, *CsFT*, *CsFTL1* and *CsMFT* were located on chromosome 8, and the other three CsPEBPs (*CsTFL1*, *CsFTL3* and *CsFTL2*) were located on chromosomes 7, 11 and 17, respectively (Figure 3a).

To examine the collinearity of PEBP genes across species, this study analyzed synteny between *C. sinense* and five representative species, including four monocots (*C. ensifolium*, *P. equestris*, *A. shenzhenica*, and *O. sativa*) and one eudicot (*A. thaliana*) (Figure 3b). Collinear regions were identified for four CsPEBPs between *C. sinense* and *C. ensifolium*, and for three between *C. sinense* and *P. equestris.* In contrast, only one *CsPEBP* was detected in syntenic blocks shared with *A. shenzhenica* or *O. sativa*. Specifically, *CsFTL2* and *CsFTL3* were present in collinear regions between *C. sinense* and both *C. ensifolium* and *P. equestris*. *CsFTL3* was also found in syntenic regions between *C. sinense* and *A. shenzhenica* or *O. sativa*. Notably, no CsPEBPs were identified in collinear regions between *C. sinense* and *A. thaliana*. Moreover, in all syntenic pairs identified, each CsPEBP corresponded to only a single ortholog in the other genome.

### 3.5. Analysis of the Cis Element of CsPEBPs Promoter

**Figure 4:**
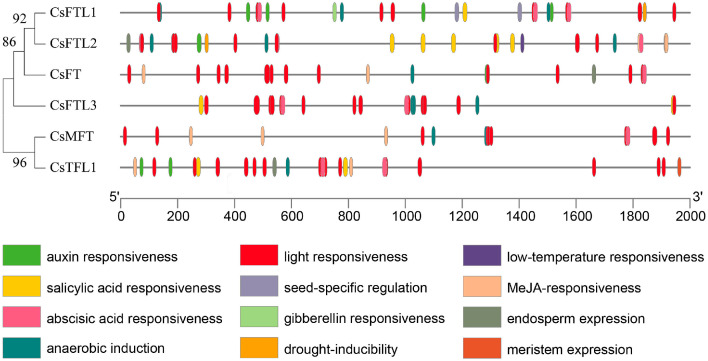
Analysis of the relative position of CERs in the promoter region of CsPEBPs. Each cis-element is represented by a different color, and its position is the same as that of the promoter.

To gain further insights into the potential functions of the genes, we analyzed the 2000 bp promoter sequences upstream of the six CsPEBPs for cis-regulatory elements (CREs) using the online tool PlantCARE. In total, 137 CREs were predicted in the promoter regions and categorized into four types: 71 light-responsive, 41 hormone-responsive, 18 stress-responsive, and 7 development-associated elements (Table S5; Figure 4). The number of hormone response elements from highest to lowest was ABA responsiveness (16), salicylic acid responsiveness (10), MeJA-responsiveness (10), auxin responsiveness (4) and GA responsiveness (1) (Figure 4). Stress-responsive elements included drought-inducibility, low-temperature responsiveness and anaerobic induction elements (Figure 4). Notably, several CREs were predicted to exist in all six *CsPEBP* promoters (Table S5; Figure 4), such as the light-responsive G-box and Box 4, the anaerobic response-related ARE, and the ABA-responsive ABRE. These results suggested that the expression of CsPEBPs may be co-regulated by the environment and corresponding hormone signals.

### 3.6. Expression Patterns of CsPEBPs Validated by qRT-PCR

**Figure 5:**
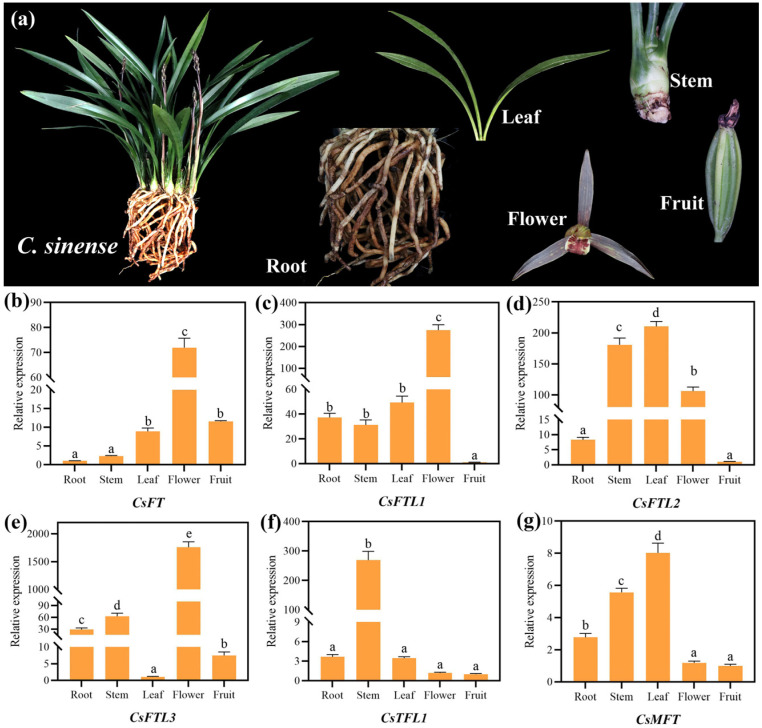
Expression analysis of CsPEBPs in different tissues of *C. sinense.* (**a**) Phenotypes of different tissues. (**b**–**g**) qRT-PCR results of six CsPEBPs in different tissues. Lowercase letters above the bar indicate the significant difference (α = 0.05, LSD) among the samples.

To clarify the expression characteristics of CsPEBPs in different organs, we first examined their expression levels in roots, stems, leaves, flowers, and fruits (Figure 5). qRT-PCR analysis revealed that the six CsPEBPs exhibited distinct tissue-specific expression patterns. Specifically, *CsFT*, *CsFTL1*, and *CsFTL3* exhibited the highest expression in *flowers*, while *CsFTL2*, *CsTFL1*, and *CsMFT* showed peak expression in leaves, stems, and leaves, respectively. These results suggest that different CsPEBPs may be involved in regulating development or physiological functions in specific organs of *C. sinense*.

**Figure 6:**
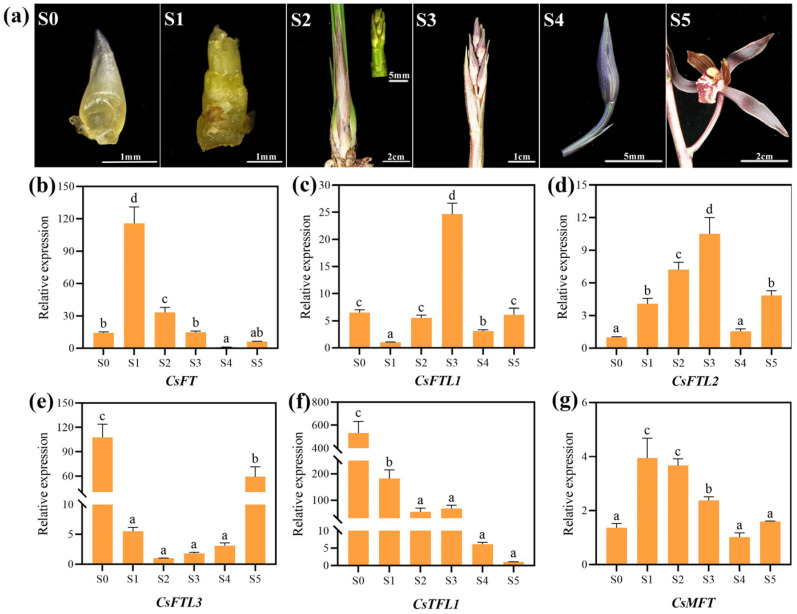
Expression analysis of CsPEBPs in different floral developmental stages of *C. sinense.* (**a**) Phenotypes of different floral developmental stages. S0: undifferentiated stage, S1: flower bud differentiation and development stage, S2: flowering stem elongation period, S3: developmental stage of immature bud arrangement, S4: advanced inflorescence maturation stage, S5: Full bloom stage. (**b**–**g**) qRT-PCR results of six CsPEBPs in different floral developmental stages. Lowercase letters above the bar indicate the significant difference (α = 0.05, LSD) among the samples.

To further elucidate the potential roles of CsPEBPs during floral bud development, we systematically analyzed their expression dynamics across six consecutive bud developmental stages (S0–S5) (Figure 6). The results showed that expression changes in these genes were closely associated with the developmental progression. The expression of *CsFT*, *CsFTL2* and *CsMFT* was low at the undifferentiated stage (S0), but was significantly upregulated at the initial differentiation stage (S1). In contrast, *CsFTL1*, *CsFTL3*, and *CsTFL1* exhibited high expression at the undifferentiated stage (S0), and decreased sharply upon the initiation of flower bud differentiation (S1). Throughout the subsequent stages of floral organ differentiation and maturation (S1–S4), the expression of *CsFTL3* and *CsTFL1* remained low or showed a continuous decline, suggesting their potential role as floral inhibitors. These expression trends corresponded well with the overall patterns initially observed in the RNA-seq-based analysis (Figure S1).

### 3.7. Cloning, Sequence Alignment and Phylogenetic Tree Analysis of CsFTL3

**Figure 7:**
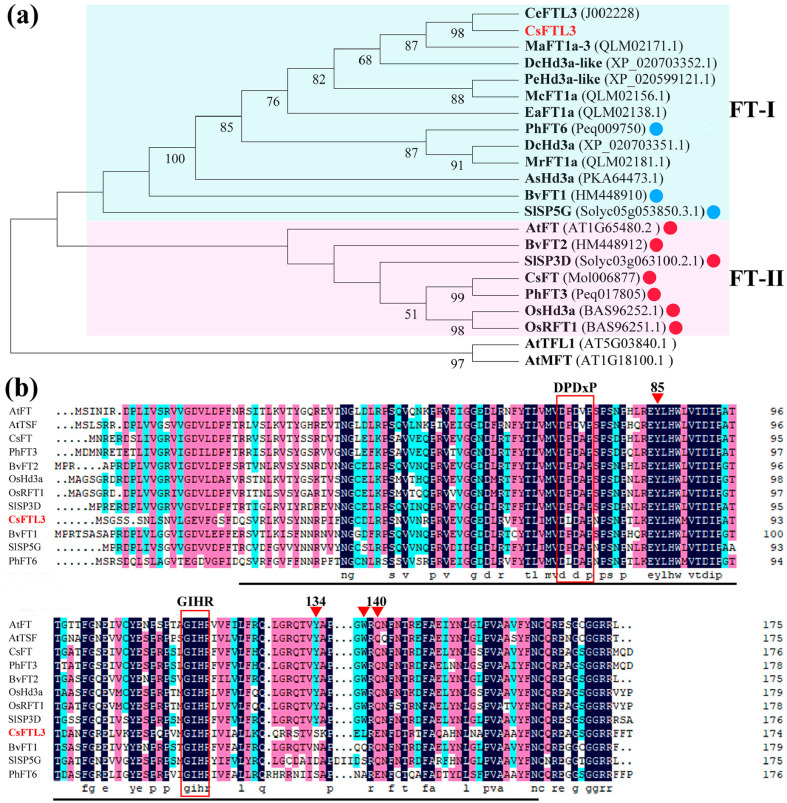
Phylogenetic tree and multiple alignment analysis of CsFTL3 protein. (**a**) Phylogenetic tree analysis of CsFTL3 and homologous proteins in other species. Blue circles represent reported FT homologous proteins that inhibit flowering, red circles represent reported FT homologous proteins that promote flowering. (**b**) Multiple sequence alignment analysis between CsFTL3 proteins and other FT homologues. The PEBP domain is represented by a black line. The red box is the conserved amino acid region. The red inverted triangles indicate the key amino acid residue sites, followed by tyrosine at position 85 (Tyr-85), tyrosine at position 134 (Tyr-134), tryptophan at position 138 (Trp-138) and glutamine at position 140 (Gln-140).

To investigate the functional evolution of CsFTL3, we cloned the gene and performed phylogenetic analysis of its protein sequence. The phylogenetic tree contained 22 representative sequences, including CsFTL3, the flowering promoter CsFT from *C. sinense*, nine functionally uncharacterized orchid proteins highly homologous to CsFTL3, several functionally identified FT homologs, and the outgroup proteins AtTFL1 and AtMFT from *A. thaliana* (Table S3). Phylogenetic analysis revealed that all FT homologs clustered into a distinct clade separate from TFL1 and MFT. Within this FT clade, CsFTL3 grouped into the FT-I branch together with known floral repressors (such as BvFT1 from sugar beet, SP5G from tomato, and PhFT6 from *Phalaenopsis*) and the nine orchid homologs. In contrast, all flowering-promoting FT homologs grouped within the FT-II branch (Figure 7a).

To further examine the sequence basis of CsFTL3 function, we performed multiple sequence alignment with typical flowering promoters (AtFT and AtTSF from *A. thaliana*, CsFT from *C. sinense*, PhFT3 from *Phalaenopsis*, BvFT2 from *Beta vulgaris*, OsHd3a and OsRFT1 from *O. sativa*, and SlSP3D from tomato) and flowering repressors (such as BvFT1 from *B. vulgaris*, PhFT6 from *Phalaenopsis*, and SlSP5G from tomato). Like other PEBP proteins, the CsPEBP members contain a highly conserved PEBP domain, comprising approximately 80% of the protein (Figure 7b), and the characteristic GIHR motif. CsFTL3 retains tyrosine only at position 85 (Tyr-85) among the key functional sites reported by Wickland and Hanzawa [15], a residue conserved in flowering promoters. In contrast, amino acid substitutions occur at three other critical positions—134, 138, and 140 (Y134S, W138L, and Q140E)—deviating significantly from the conserved pattern of typical flowering promoters (Figure 7b). Notably, the flowering repressors BvFT1, SlSP5G, and PhFT6 also retain tyrosine at position 85 but exhibit variations at positions 134 and 138. Furthermore, PhFT6 carries a mutation at position 140, identical to CsFTL3, substituting glutamine with glutamate (Glu). Together, phylogenetic placement and key residue variations indicate that although CsFTL3 evolutionarily belongs to the FT family, amino acid variations at critical positions—particularly 134, 138, and 140—likely alter protein surface properties and interaction interfaces, leading to functional divergence from flowering promotion to inhibition. This provides a molecular structural basis for its biological role as a flowering repressor.

### 3.8. Subcellular Localization of CsFTL3 Protein in C. sinense Protoplasts

**Figure 8:**
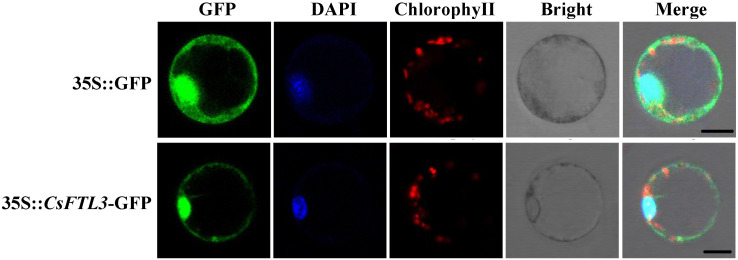
Subcellular localization of CsFTL3 in protoplasts of *C. sinense*. The GFP signals of the CsFTL3::GFP fusion proteins appeared in the nucleus, cell membrane and cytoplasm. The free GFP was driven by the 35S promoter as a control. Bar = 50 μm.

Subcellular localization was predicted for CsFTL3 using Cell-PLoc, which indicated a nucleus location. To validate this prediction, we constructed a PAN580-*CsFTL3*::GFP fusion vector and transiently expressed it, along with a GFP-alone control, in petal-derived protoplasts of *C. sinense* ‘Xiao Xiang’. The results of subcellular localization showed that, like the empty vector control, CsFTL3 was localized in the nucleus, cell membrane and cytoplasm (Figure 8). The observed localization pattern, which extends beyond the predicted nuclear confinement, may arise from several technical or biological factors. These include the transient overexpression of the *CsFTL3*::GFP fusion vector, potential artifacts from the GFP tag, or the possibility that CsFTL3 undergoes dynamic nucleocytoplasmic shuttling in planta. A definitive interpretation requires further investigation.

### 3.9. Overexpression of CsFTL3 in Arabidopsis Affected Flowering Time

**Figure 9:**
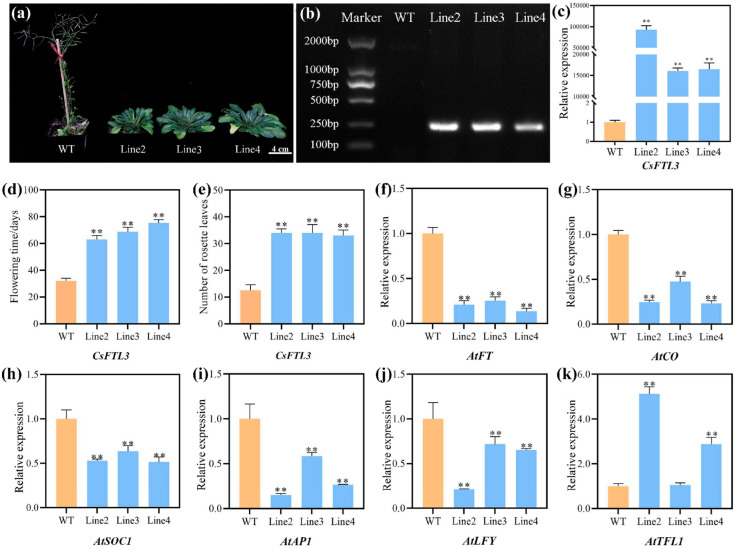
Heterologous overexpression of *CsFTL3* in *Arabidopsis* verified its role in flowering time. (**a**) Delayed flowering of transgenic *Arabidopsis* overexpressing *CsFTL3*. (**b**) Verification of transgenic plants by agarose gel electrophoresis. (**c**) Analysis of expression level of *CsFTL3* in transgenic plants. (**d**) Statistical analysis of flowering time. (**e**) Statistical analysis of the number of rosette leaves. (**f**–**k**) Expression level of flowering-related genes in *Arabidopsis*. Asterisks indicate significant differences: **, *p* < 0.01.

*CsFTL3* was highly expressed at the undifferentiated stage of flower bud development but declined sharply upon differentiation and remained low thereafter. To validate its role in flowering regulation, we introduced the recombinant vector pOCA30-*CsFTL3*, which contains the *CsFTL3* coding sequence driven by the CaMV 35S promoter, into wild-type (WT) *Arabidopsis* (Col-0). A total of 27 transgenic lines were obtained through subsequent antibiotic selection. Three randomly selected lines (Line2, Line3, and Line4) were confirmed as PCR-positive (Figure 9b), and qRT-PCR results showed significantly high expression of *CsFTL3* in these lines (Figure 9c). Homozygous T3 transgenic plants from these three lines were used for further analysis. Compared with WT, transgenic *Arabidopsis* lines exhibited significantly delayed flowering and a marked increase in rosette leaf number (Figure 9a). The flowering time of WT was 32.0 days, while those of the overexpressing lines Line2, Line3, and Line4 were 63.0, 68.7, and 75.3 days, respectively (Figure 9d). The corresponding rosette leaf numbers were 12.6 (WT), 34.5 (Line2), 34.0 (Line3), and 33.0 (Line4). These results demonstrate that *CsFTL3* inhibits flowering and promotes vegetative growth in *Arabidopsis*.

To examine whether *CsFTL3* influences flowering-related gene expression in *Arabidopsis*, we analyzed the expression of *AtFT*, *AtCO*, *AtSOC1*, *AtAP1*, *AtLFY*, and *AtTFL1*. Among these, *AtFT*, *AtCO*, *AtSOC1*, *AtAP1*, and *AtLFY* were significantly downregulated in all three overexpressing *CsFTL3* lines compared to the WT, whereas *AtTFL1* was upregulated in Line2 and Line4, but not significantly altered in Line3.

### 3.10. Validation of CsFTL3 Function in C. sinense by VIGS

**Figure 10:**
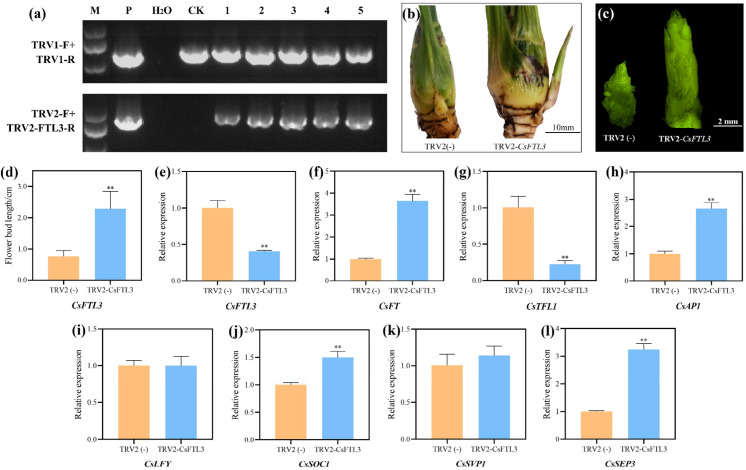
VIGS validated the role of the *CsFTL3* in regulating flowering time in *C. sinense*. (**a**) PCR identification of TRV2-*CsFTL3* plants. M, P, H_2_O, and CK represent the DNA marker, positive plasmid, water, and empty TRV2 plants, respectively. 1–5 represent the TRV2-*CsFTL3* plants. (**b**,**c**) Phenotypic comparison of floral buds between TRV2- and TRV2-*CsFTL3*-silenced *C. sinense* plants. (**d**) Statistics of floral bud length. (**e**) Silencing efficiency of *CsFTL3* detected by qRT-PCR. (**f**–**l**) Expression levels of flowering-related genes. Asterisks indicate significant differences: **, *p* < 0.01.

To elucidate the function of *CsFTL3* in the floral transition of *C*. *sinense*, we employed VIGS to knock down its expression. Successful VIGS infection was confirmed by PCR, with positive plants showing amplification of a band consistent with the expected size (Figure 10a). qRT-PCR analysis revealed that the expression level of *CsFTL3* in floral buds of TRV2-*CsFTL3* plants was significantly lower compared to the empty TRV2 vector control, indicating effective gene silencing (Figure 10e). Upon *CsFTL3* silencing, phenotypic observations showed accelerated floral bud development. The floral buds of *CsFTL3*-silenced plants were significantly longer than those of the control group (Figure 10b,d). Furthermore, the floral buds of silenced plants had advanced to the floret primordia differentiation stage, whereas most control plants remained at the inflorescence primordia differentiation stage or undifferentiated state (Figure 10c). Further molecular analysis demonstrated that in silenced plants, the expression of key flowering-promoting genes (*CsFT*, *CsAP*1, *CsSOC1*, and *CsSEP3*) was significantly upregulated (Figure 10f,h,j,l), whereas the flowering repressor *CsTFL1* was significantly downregulated (Figure 10g). No significant changes were observed in the expression of *CsLFY* and *CsSVP1* (Figure 10i,k). These results indicate that silencing *CsFTL3* promotes floral transition and accelerates floral bud development in *C*. *sinense*.

### 3.11. Protein–Protein Interaction (PPI) Network Analysis of CsFT and CsFTL3

**Figure 11:**
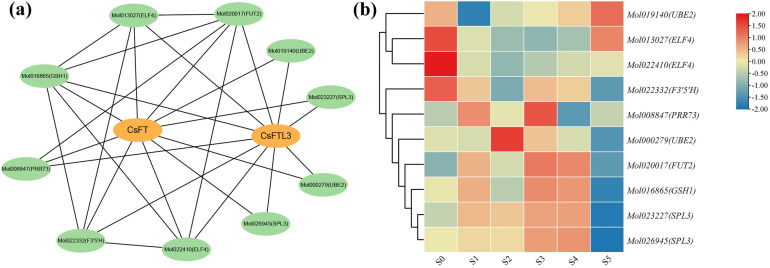
Prediction of interaction proteins between CsFT and CsFTL3. (**a**) The PPI network of CsFT and CsFTL3. Each ellipse represents a protein, and lines connect interacting protein pairs. (**b**) FPKM values of genes encoding the predicted interacting proteins across different flower bud development stages. The color gradient from blue to red represents relative abundance based on FPKM, from low to high.

Based on homology to known interacting proteins from model plants, we predicted the PPI network of CsFT and CsFTL3 (Figure 11a). The 10 predicted interacting partners included two ubiquitin-conjugating enzymes (UBE2), two early flowering proteins (ELF4), two SPL3 transcription factors (TFs), and one each of flavonoid 3′,5′-hydroxylase (F3′5′H), a CCT gene family protein (PRR73), galactoside 2-α-L-fucosyltransferase (FUT2), and glutamate-cysteine ligase (GSH1). These predicted interacting partners are implicated in many key biological processes, including ubiquitination, photoperiodic flowering regulation, transcriptional control of flower development, flavonoid biosynthesis, circadian rhythms, glycosylation, and antioxidant metabolism, suggesting potential roles for CsFT and CsFTL3 in these regulatory networks.

Based on transcriptome data, the coding genes of these putative interacting partners displayed dynamic FPKM profiles across flower bud developmental stages (Figure 11b), revealing their dynamic expression patterns. For instance, transcripts of two *ELF4* homologs and the *UBE2* (Mol019140) were more abundant in the undifferentiated stage (S0) according to FPKM values, but their levels appeared lower at the transition stage (S1). In contrast, FPKM values indicated increased abundance of *PRR73* and *SPL3* during differentiation. These observed trends, together with the PPI predictions, provide preliminary clues that CsFT and CsFTL3 may interact with distinct sets of partners at different stages to regulate flowering.

### 3.12. Cloning of CsFTL3 Promoter and Identification of Its Upstream Potential TFs

**Figure 12:**
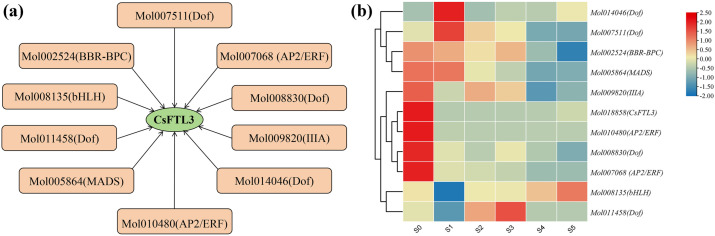
Bioinformatic analysis of the putative transcriptional regulatory network of *CsFTL3*. (**a**) Predicted network of upstream TFs. The beige square and green ellipse represent candidate upstream TFs and *CsFTL3*, respectively. (**b**) FPKM values of the predicted upstream TF genes across different flower bud development stages. The color gradient (blue to red) represents relative transcript abundance based on FPKM, from low to high.

In this study, we cloned a 2000 bp promoter region upstream of the flowering repressor gene *CsFTL3* and performed bioinformatic analysis to identify potential upstream TFs, to elucidate its regulatory mechanism. Using comparative genomic and homology analyses with the genomes of *A. thaliana*, *P. equestris*, and *O. sativa*, we constructed a putative transcriptional regulatory network upstream of *CsFTL3* in *C. sinense* (Figure 12a). Ten TFs were predicted as potential regulators, including four Dof, two AP2/ERF, and one each of BBR-BPC, bHLH, IIIA, and MADS-box TFs. The prevalence of Dof family members among the candidates suggests their potential importance.

We further examined the FPKM profiles of these predicted TFs across different flower bud developmental stages (Figure 12b). The patterns suggested that multiple TFs might temporally regulate *CsFTL3*. Among them, AP2/ERF (Mol007068 and Mol010480) and Dof (Mol008830) displayed higher FPKM values during the undifferentiated stage (S0), which decreased at the initial differentiation stage (S1) and remained relatively low thereafter. This pattern was similar to that of *CsFTL3*, suggesting these TFs are candidate activators maintaining *CsFTL3* expression at S0 to prevent premature differentiation. In contrast, Dof (Mol014046 and Mol007511) exhibited an opposite trend, with lower FPKM at S0 and increased values at S1, suggesting a potential role in repressing *CsFTL3* transcription to release floral inhibition upon initiation.

### 3.13. Effects of Abiotic Stress on the Expression of CsFTL3

**Figure 13:**
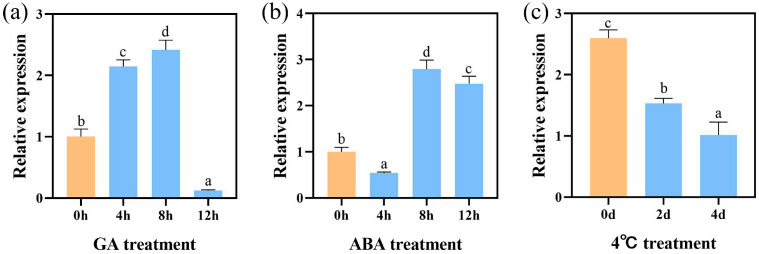
Effects of hormone and low temperature treatment on the expression of *CsFTL3.* (**a**) GA treatment. (**b**) ABA treatment. (**c**) 4 °C treatment. Lowercase letters above the bar indicated the significant difference (α = 0.05, LSD) among the samples.

To investigate the effects of abiotic stress on the expression of *CsFTL3* in *C. sinense*, we analyzed its expression patterns under treatments of exogenous GA, ABA, and low temperature (4 °C). qRT-PCR results showed that all treatments significantly altered *CsFTL3* expression (Figure 13). As shown in Figure 13a, GA transiently induced *CsFTL3* expression, with transcript levels increasing at 4 h and 8 h but declining sharply by 12 h to below the initial (0 h) control level. This dynamic expression pattern showed that GA treatment was accompanied by a rapid downregulation of *CsFTL3* transcripts, which coincided temporally with the promotion of floral transition. Under ABA treatment (Figure 13b), *CsFTL3* expression was slightly suppressed at 4 h, significantly induced at 8 h, and remained elevated at 12 h compared to the control. This upregulation coincided with the known role of ABA in delaying flowering. In contrast, low temperature (4 °C) strongly suppressed *CsFTL3* expression (Figure 13c), with a marked reduction after 2 d and a further decrease by 4 d. This sustained downregulation under cold stress is consistent with the well-established phenomenon of low temperature-induced floral transition in many plants. Together, these results suggest that the multi-hormonal responsiveness of *CsFTL3* positions it as a potential integrator of environmental signals, aligning with the emerging paradigm that floral development is orchestrated by dynamic shifts in complex hormone signaling networks [54].

## 4. Discussion

Genes of the PEBP family play key regulatory roles in higher plants, regulating processes such as floral transition, seed development and dormancy, and inflorescence architecture formation [55,56,57]. To date, PEBP family genes have been identified at the genome-wide level in various plant species, including rice [21], tomato [24], and *C. mollissima* [28]. In this study, we identified six PEBP family genes from the *C. sinense* genome database and designated them as *CsFT*, *CsFTL1*, *CsFTL2*, *CsFTL3*, *CsTFL1*, and *CsMFT* (Table 1). The number of PEBP genes in *C. sinense* is lower than in rice (19), *M. integrifolia* (13), and tomato (12), but comparable to *Arabidopsis* (6), *D. huoshanense* (6), and *P. mume* (5) [21,24,27,31,35,58]. Variation in the PEBP gene copy number across species may be due to gene duplication or loss events during evolution. Nevertheless, monocots generally possess more PEBP members than eudicots (Figure 1b). Similarly to *Arabidopsis*, rice, and tomato, the PEBP genes in the *C. sinense* genome can be classified into three subfamilies: *FT*-like (*CsFT*, *CsFTL1*, *CsFTL2*, and *CsFTL3*), *TFL1*-like (*CsTFL1*), and *MFT*-like (*CsMFT*) (Figure 1a). This classification is consistent with findings in other ornamental plants such as *C. ensifolium*, *P. mume*, and lotus [31,37,59]. Notably, the *FT*-like subfamily is predominant in *C. sinense* (4/6), as is common in monocots such as rice, potentially associated with specific environmental selection pressures and unique life history strategies during monocot evolution [60,61].

Previous studies indicate that variation in exon-intron structure plays a role in plant evolution [62]. In this study, most CsPEBPs shared a conserved gene structure of four exons and three introns (Figure 2b), consistent with the high conservation of PEBP genes across species [24,31] and reflecting their evolutionary conservation. By contrast, *CsFTL1* and *CsFTL2* within the *FT*-like subfamily contained five longer introns (Figure 2b). Such variations in intron number and length may influence transcriptional efficiency, mRNA stability, and the occurrence of alternative splicing, and this could provide a mechanism for functional diversification within the *FT*-like subfamily in *C. sinense* [63]. Conserved motif analysis revealed that all CsPEBPs except CsMFT contained Motif1 through Motif4 arranged in the same order, indicating ancient conservation of the functional core region. The unique presence of Motif5 and Motif6 in CsMFT, as well as Motif5 in CsFTL1 and Motif6 in CsFTL2, may underlie distinct regulatory functions or interaction specificities to these members [28].

Collinearity analysis is commonly used to reveal genetic relationships within or between species. Although the *C. sinense* genome has undergone extensive rearrangements and exhibits low collinearity with distantly related species [2], our analysis still revealed both conservation and specificity in the evolution of its PEBP gene family. Notably, *CsFTL3* retained orthologs across several monocots, including *C. ensifolium*, *P. equestris*, *A. shenzhenica*, and *O. sativa*, indicating high evolutionary conservation of this gene in the PEBP family (Figure 3b). In contrast, and consistent with reports in *D. huoshanense* and *A. thaliana* [35], we detected no CsPEBPs in collinear regions with *A. thaliana*, suggesting that their evolutionary trajectory in *C. sinense* may be independent of that in *A. thaliana*, with its expansion and functional diversification likely occurring specifically within the orchid lineage. Furthermore, each CsPEBP identified in collinear regions with other species corresponded to only a single ortholog. According to the gene balance hypothesis [64], this pattern suggests these CsPEBPs have undergone high functional specialization and are released from strict dosage constraints. This finding provides a new evolutionary perspective for understanding the complex functional differentiation of the PEBP family in orchids, suggesting that these genes may play specialized roles in orchid-specific physiological processes, such as flowering time regulation and floral morphogenesis.

Our analysis of the CsPEBPs promoters revealed a diverse array of CREs, which are crucial for controlling gene expression. A notable enrichment of light- and hormone-responsive elements was observed (Figure 4), which is consistent with the well-established role of PEBP genes in integrating environmental and hormonal cues to regulate floral development in plants [65]. For instance, the promoters of *CsFT* and *CsTFL1* contain multiple CREs associated with light and hormone responses (Figure 4), suggesting that their transcription may be co-regulated by endogenous hormones (e.g., GA and ABA) and external factors such as photoperiod to modulate floral transition [42,66]. Indeed, their expression profiles exhibit stage-specific shifts during the vegetative-to-reproductive transition (Figure 6), supporting this regulatory paradigm. Therefore, in-depth functional characterization of CsPEBPs will facilitate precise control of flowering time in *C. sinense*.

Gene expression patterns are often closely associated with their biological functions. Our qRT-PCR analysis revealed distinct organ-specific expression profiles for the six CsPEBPs (Figure 5), supporting their involvement in diverse aspects of *C. sinense* development. Notably, *CsFTL3* showed the highest expression in floral organs (Figure 5), a spatial expression pattern similar to that reported for *AcFT3*/*AcFT4* in pineapple [25] and for *FT*-like genes in *Vigna radiata* [67]. More critically, the temporal expression dynamics of *CsFT* and *CsFTL3* during floral transition were clearly antagonistic (Figure 6). *CsFT* transcripts were significantly induced at the initial differentiation stage (S1), which is fully consistent with its established role as a florigenic activator [15] and our prior functional validation in *Arabidopsis* [38]. Conversely, *CsFTL3* was highly expressed at the undifferentiated stage (S0) but sharply declined upon floral initiation (S1), with its expression remaining low during subsequent development (Figure 6). This precise inverse correlation with the commitment to flowering suggests that *CsFTL3* functions as a floral repressor analogous to *TFL1*, preventing premature transition.

Across plant species, *FT* typically possesses a conserved flowering-promotion function, as exemplified by *Hd3a* in rice, *SFT* in tomato, and *ZCN8* in maize [15]. However, we identified *CsFTL3*, an *FT* homolog that functions as a flowering repressor. Consistently, heterologous overexpression in *Arabidopsis* significantly delayed flowering and altered the expression patterns of key flowering-related genes (Figure 9), while silencing in *C. sinense* accelerated flower bud development, and similarly modified the expression profiles of flowering-related genes (Figure 10). This finding aligns with reports of other repressive *FT*-like genes, including *HvFT4* in barley, which delays flowering under long-day conditions [68]; *PhFT6* in *Phalaenopsis,* whose heterologous expression inhibits flowering in *Arabidopsis* [34]; and *OsFTL12* in rice, which delays heading by forming a floral repression complex [69]. The functional specificity of FT homologs often stems from variations in a few critical amino acid residues [70]. Phylogenetic analysis placed CsFTL3 within the FT-I alongside known floral repressors, and specific variations were identified at key functional sites, suggesting potential functional divergence during evolution (Figure 7). We observed critical amino acid substitutions in CsFTL3 (Y134S, W138L, and Q140E) similar to those in PhFT6, further supporting its repressive function. These substitutions may alter protein surface properties and interaction interfaces, affecting specificity with downstream signaling components and ultimately leading to functional divergence.

Beyond sequence divergence, the regulatory role of *CsFTL3* is likely embedded in a multi-layered network. Our homology-based in silico analyses offer a preliminary view of this complexity. First, the predicted PPI network suggests that CsFTL3 (and CsFT) may function through interactions with partners involved in ubiquitination, photoperiod response, and transcriptional control (Figure 11a). The stage-specific expression patterns of these putative partners (Figure 11b) support, but do not prove, a model of dynamically assembled complexes during development. Second, predicted upstream regulators, including Dof and AP2/ERF family TFs, point to transcriptional control (Figure 12). Their antagonistic expression patterns suggest a “push-pull” module fine-tuning *CsFTL3* expression at the critical floral transition stage. Additionally, seasonal flowering in *C. sinense* is thought to be associated with the regulation of *SVP* in response to low temperature [43]. Similarly, in *Phalaenopsis*, *PhSVP* represses *PhFT6* expression by directly binding to CArG elements in its promoter, thereby promoting earlier flowering [34]. Notably, among our predicted regulatory factors, a MADS-box transcription factor (Mol005864) emerged as a candidate for an *SVP*-like regulatory mechanism of *CsFTL3*. Collectively, these predictions position *CsFTL3* as a candidate hub in a broader regulatory system. Future experimental work, such as yeast two-hybrid and chromatin immunoprecipitation assays, is needed to validate these interactions and solidify the network model.

## 5. Conclusions

We performed a genome-wide identification and functional analysis of the PEBP gene family in *C. sinense* and identified *CsFTL3* as a key floral inhibitor, a conclusion robustly supported by both gain- and loss-of-function experiments. Expression analysis showed that *CsFTL3* is highly expressed prior to floral commitment but declines sharply upon initiation, a pattern opposite to the flowering promoter *CsFT*. Consistent with this repressive expression pattern, heterologous overexpression in *Arabidopsis* delayed flowering, whereas VIGS-mediated knockdown in *C. sinense* accelerated floral development, establishing *CsFTL3* as a major genetic brake on the reproductive transition. Phylogenetically, *CsFTL3* clusters within the repressive FT-I clade, and its expression is modulated by hormones and low temperature, positioning it as an integrator of environmental and developmental cues. Furthermore, in silico analyses suggest it operates within a complex regulatory network, though future work is needed to validate the predicted molecular interactions. Collectively, this work establishes *CsFTL3* as a crucial regulator of flowering time in orchids, thereby providing both a precise molecular target and a theoretical foundation for molecular breeding aimed at predictable flowering control and year-round production.

## Figures and Tables

**Table 1:** Details of the six PEBP proteins in *C. sinense*.

Name	Gene Model ID	gDNA (bp)	CDS Length (bp)	Peptide Residue (AA)	Molecular Weight (MW)	Instability Index	Aliphatic Index	GRAVY	PI	Predicted Subcellular Localization
CsFT	Mol006877	2989	531	176	19,848.39	43.37	80.74	−0.311	6.42	Nucleus
CsFTL1	Mol006012	10,975	537	178	19,979.67	45.09	80.96	−0.335	5.93	Nucleus
CsFTL2	Mol017362	18,869	546	181	20,628.44	43.85	75.36	−0.383	7.67	Nucleus
CsFTL3	Mol018858	4222	525	174	19,523.09	48.29	77.82	−0.375	9.03	Nucleus
CsMFT	Mol020542	3867	441	146	16,495.97	63.67	73.42	−0.385	9.04	Cytoplasm
CsTFL1	Mol012752	929	522	173	19,607.53	45.42	82.14	−0.202	9.06	Nucleus

## Data Availability

The original contributions presented in the study are included in the article and Supplementary Material, further inquiries can be directed to the corresponding authors.

## Supplementary Materials

The following supporting information can be downloaded at: https://www.mdpi.com/article/10.3390/plants15020252/s1, Figure S1: Expression profile of CsPEBPs. (**a**) Expression patterns of 6 CsPEBPs in different organs. (**b**) Expression levels of 6 CsPEBPs in 6 different flower bud development stages (S0–S5). S0: undifferentiated stage, S1: flower bud differentiation and development stage, S2: flowering stem elongation period, S3: developmental stage of immature bud arrangement, S4: advanced inflorescence maturation stage, S5: Full bloom stage. The red and blue bars (FPKM) indicate high and low expression, respectively. Table S1: Information on PEBP proteins from 16 species used for phylogenetic analysis. Table S2: Primer sequence information used in this study. Table S3: Protein information used for phylogenetic analysis and multiple sequence alignment of CsFTL3. Table S4: Statistics of three types of PEBP proteins in 16 plant species. Table S5: Prediction statistics of CREs in the promoter of CsPEBPs in *C. sinense*.
