# Supplementary material for: Genome-Wide Identification and Expression Analysis of the PEBP Gene Family in Cymbidium sinense Reveals CsFTL3 as a Floral Inhibitor
Source: Plants (Basel). 2026 Jan 13;15(2):252. doi: 10.3390/plants15020252 (PMC12845063; doi:10.3390/plants15020252)
Supplement: Supplementary file 1 [file plants-15-00252-s001.zip › Fig. S1.pdf]

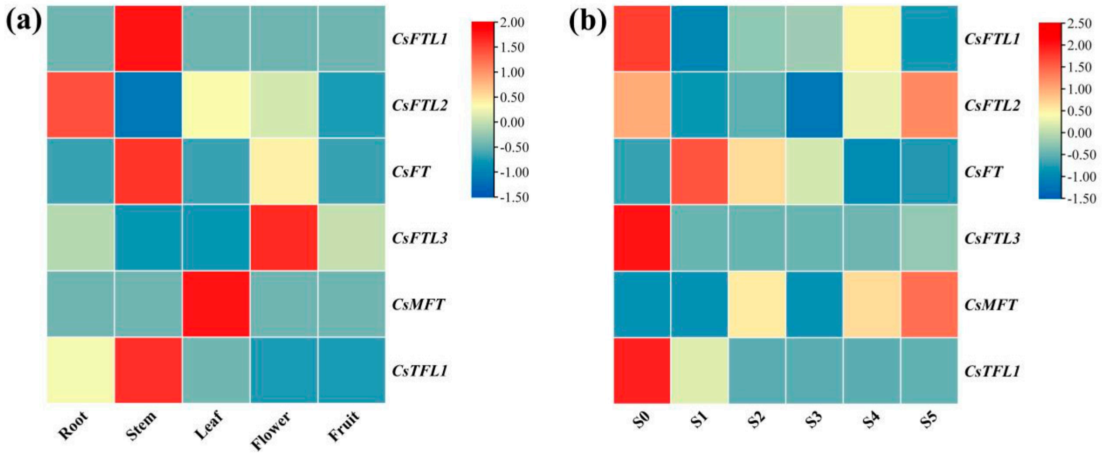

Figure S1. Expression profile of *CsPEBPs*. (a) Expression patterns of 6 *CsPEBPs* in different organs. (b) Expression levels of 6 *CsPEBPs* in 6 different flower bud development stages (S0-S5). S0: undifferentiated stage, S1: flower bud differentiation and development stage, S2: flowering stem elongation period, S3: developmental stage of immature bud arrangement, S4: advanced inflorescence maturation stage, S5: Full bloom stage. The red and blue bars (FPKM) indicate high and low expression, respectively.
