# Supplementary figures and images for: Genome-Wide Identification and Expression Analysis of the PEBP Gene Family in Cymbidium sinense Reveals CsFTL3 as a Floral Inhibitor
Source: Plants (Basel). 2026 Jan 13;15(2):252. doi: 10.3390/plants15020252 (PMC12845063; doi:10.3390/plants15020252)

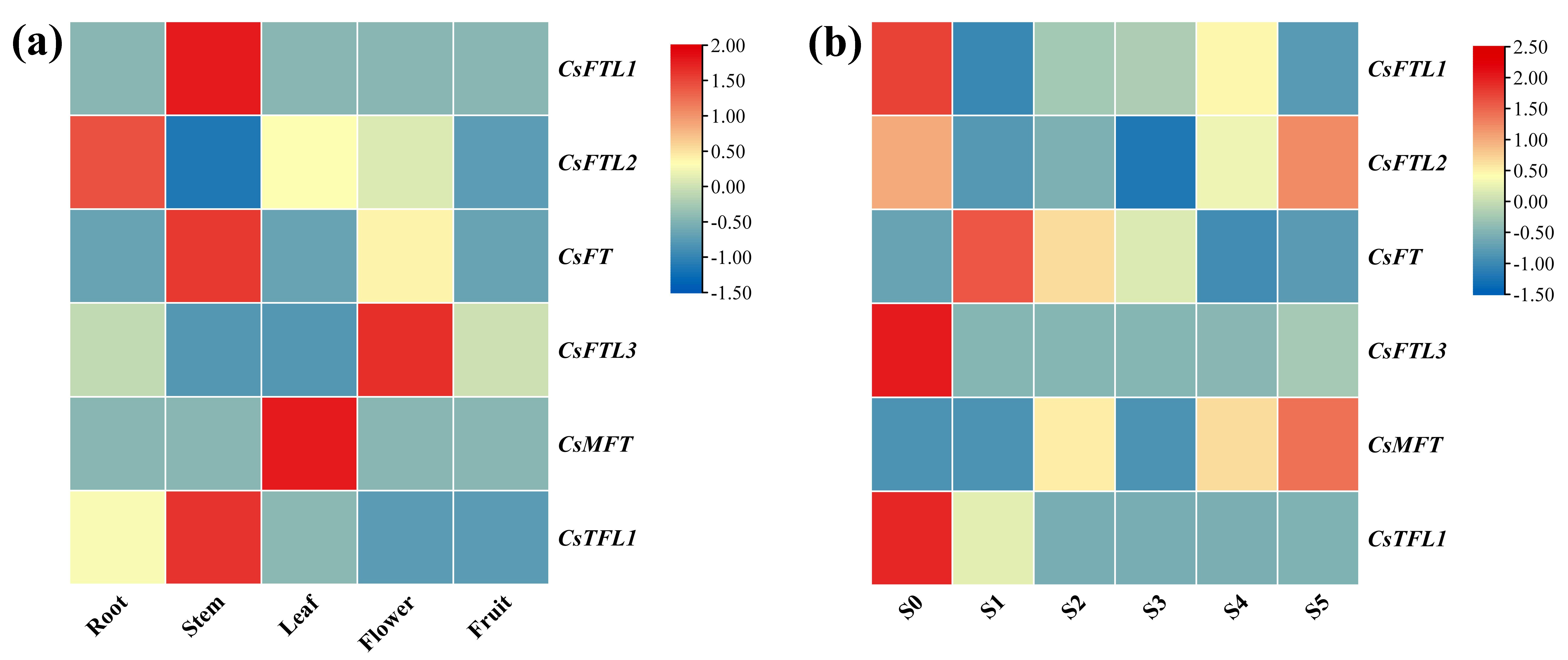

Supplement: Supplementary file 1 [file plants-15-00252-s001.zip › Fig. S1.jpg]
